# Superthermal Al Atoms as a Reactive-Atom Probe of Fluorinated Surfaces

**DOI:** 10.1021/acs.jpca.3c02167

**Published:** 2023-06-23

**Authors:** Paul D. Lane, Thomas Gstir, Simon M. Purcell, Michal Swierczewski, Naomi S. Elstone, Duncan W. Bruce, John M. Slattery, Matthew L. Costen, Kenneth G. McKendrick

**Affiliations:** † Institute of Chemical Sciences, School of Engineering and Physical Sciences, Heriot-Watt University, Edinburgh EH14 4AS, U.K.; ‡ Institut für Ionenphysik und Angewandte Physik, Universität Innsbruck, Innsbruck 6020, Austria; § Department of Chemistry, University of York, Heslington, York YO10 5DD, U.K.

## Abstract

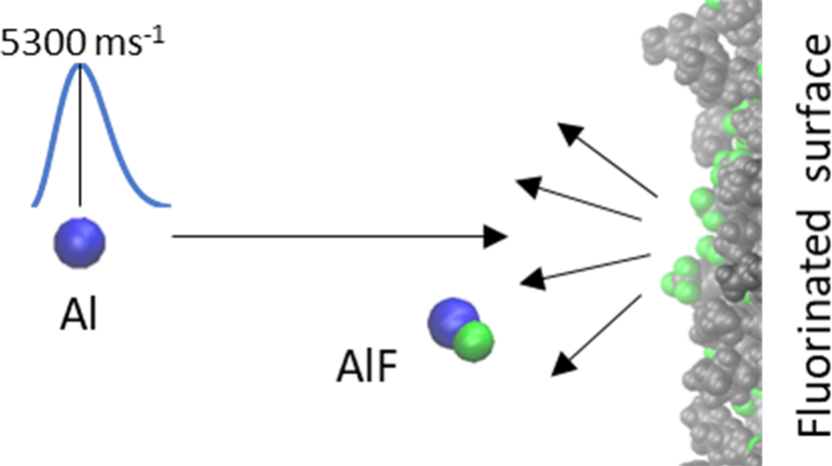

We demonstrate a proof-of-concept of a new analytical technique to measure relative F atom exposure at the surfaces of fluorinated materials. The method is based on reactive-atom scattering (RAS) of Al atoms, produced by pulsed laser ablation of solid Al at 532 nm. The properties of the incident ground-state Al were characterized by laser-induced fluorescence (LIF); at typical ablation fluences, the speed distribution is approximately Maxwellian at ∼45000 K, with a most-probable kinetic energy of 187 kJ mol^–1^ and a mean of 560 kJ mol^–1^ When these Al atoms impact the surfaces of perfluorinated solids (poly(tetrafluorethylene), PTFE) or liquids (perfluoropolyether, PFPE), gas-phase AlF products are clearly detectable by LIF on the AlF A–X band. Quantitative AlF yields were compared for a small representative set of a widely studied family of ionic liquids based on the common 1-alkyl-3-methylimidazolium ([C_*n*_mim]^+^) cation. Yields of (1.9 ± 0.2):1 were found from [C_2_mim][Tf_2_N] and [C_8_mim][Tf_2_N], containing the common fluorinated bis(trifluoromethylsulfonyl)imide anion ([Tf_2_N]^−^). This is in quantitative agreement with previous independent low-energy ion scattering (LEIS) measurements and consistent with other independent results indicating that the longer cationic alkyl chains cover a larger fraction of the liquid surface and hence reduce anion exposure. The expected null result was obtained for the ionic liquid [C_2_mim][EtSO_4_] which contains no fluorine. These results open the way for further characterization and the potential application of this new variant of the RAS-LIF method.

## Introduction

The gas–liquid interface is central to many processes of practical interest, including gas separation and sequestration, biological respiration, heterogeneous reactions in the atmosphere, and forms of multiphase catalysis. As a result, there is a great desire to be able to probe the composition and structure of the surfaces of liquids.

A wide range of experimental techniques have been developed for this purpose, but they differ fundamentally in the depths to which they probe and hence what constitutes “the surface” for the purposes of the measurement. Our interest here is in identifying components of the liquid that are directly exposed to impacts with molecules arriving from the gas phase. Although the liquids of practical interest in applications of the type noted above are very diverse, most information is, perhaps understandably, known about those amenable to the widest array of experimental methods. Because many of the techniques rely on large gas-phase mean free paths for ions, molecules, or electrons either directed at the surface or detected after being emitted from it, the most-studied liquids have necessarily been those with very low vapor pressures. One particularly interesting class of molecules in this category, ionic liquids (ILs), will feature prominently in the developments we present here. We emphasize, though, that the new technique we present is not confined to the study of materials of this specific type.

The surfaces of ILs can, of course, be probed by classical methods such as surface tension, which offer an indirect measure of the surface composition. Advanced techniques, including neutron reflectometry (NR)^1^ and X-ray reflectometry (XRR),^2^ provide information on scattering length as a function of depth from which chemical composition can also be inferred through modeling. Alternative methods have been applied that probe the composition more directly, including angle-resolved photoelectron spectroscopy (ARXPS),^3−10^ high-resolution Rutherford backscattering (HRBS),^11−13^ low-energy ion scattering (LEIS),^14−16^ metastable atom electron spectroscopy (MAES),^17,18^ neutral impact collision ion scattering (NICISS),^3,19−22^ and direct recoil spectroscopy (DRS).^23,24^ An important complement has been provided by optical nonlinear surface spectroscopies such as second harmonic generation (SHG) and sum frequency generation (SFG).^2,25−28^ There are advantages and disadvantages to each of these techniques, not only in their penetration depths but also, most notably, in their chemical specificity. This has often made drawing quantitative comparisons between different techniques difficult, and so this field remains one of active inquiry.

Our approach to the study of liquid surfaces has been the use of reactive-atom scattering (RAS) techniques. These allow direct determination of the surface composition by measuring relative yields of a specific, detectable gas-phase product that results from reaction of an incoming probe atom with a specific type of functional group exposed at the surface. In the majority of this work so far, O(^3^P) atoms have been used as the probe and the products of reactions with aliphatic groups at the liquid surface detected in the gas phase. These experiments have either used laser-induced fluorescence (RAS-LIF)^29−36^ to detect the OH products or mass spectrometry (RAS-MS)^32,35,37−39^ to detect the OH and H_2_O products. The RAS-LIF approach grew out of fundamental studies of reactions of photolytically produced, moderately superthermal O(^3^P) probe atoms with the surfaces of squalane (2,6,10,15,19,23-hexamethyltetracosane),^40^ other long-chain hydrocarbons,^41^ and self-assembled monolayers (SAMs).^42,43^ It has subsequently been applied extensively to IL systems.^29,32−34,36,38,39,44^ Higher-energy O(^3^P) atoms from a hyperthermal source were used in Minton’s pioneering studies of reactions with liquid squalane,^45−47^ which became the basis of the RAS-MS method applied to related ILs.^32,35−39^ More recently, the RAS-MS approach has also been extended to using hyperthermal F atoms to abstract H or D atoms from isotopically labeled ILs.^39^

The types of information on IL systems derived from these RAS techniques include *direct* measures of increases of surface alkyl coverage with cationic alkyl chain length,^32,34,36,38^ the effect of the anion volume on cationic alkyl-chain exposure as a function of its length,^32^ and the surface enrichment of [C_12_mim]^+^ ions in mixtures of [C_2_mim]^+^ and [C_12_mim]^+^ (where [C_*n*_mim] represents a 1-alkyl-3-methylimidazolium cation with alkyl chain length, *n*).^29^ We have also inferred *indirectly*, by measuring lower-than-statistical exposure of the alkyl component, that fluoroalkyl cations have a greater surface affinity than the alkyl cations in alkyl/fluoroalkyl IL mixtures.^44^

The goal of the new experiments presented here is a step toward greater variety in the functional groups that can be targeted by RAS measurements through the abstraction of other types of atoms, thus measuring surface exposures of interest *directly*. The requirements to achieve this are a sufficiently energetic source of a suitable probe atom, a reaction pathway for abstraction of the desired target, and a chemically specific means of detection.

Motivated by this general goal and in part by our own interest in fluorinated surfaces, we sought to identify suitable probe-atom candidates for F atom detection based on their thermochemistry and spectroscopy. The thermochemistry immediately points toward metal-atom probes. The product specificity in RAS-LIF comes from the LIF detection, which excludes group 1 metals, because their diatomic metal fluoride molecules lack bound excited states in accessible wavelength regions. Group 2 metals are a possibility that remains to be explored, but in this work, we focus on the group 13 metals. They are particularly promising candidates because formation of the diatomic metal fluoride products is very thermodynamically favored; this is apparent by considering that the simplest member, BF, is isoelectronic with N_2_. However, BF does not have probe transitions in a convenient wavelength region, nor is atomic B easy to generate.

For these reasons, we selected to investigate reactions of Al atoms. The AlF bond is among the strongest known, with an experimental bond energy of ∼675 kJ mol^–1^ that is closely matched by the most recent high-level calculations.^48^ Reactions with almost all F-containing species will be exothermic, including e.g. fluoropolymers or fluoroalkyl substituents, in which C–F bond strengths will be similar to the known values of ∼530 kJ mol^–1^ for simple fluoroalkanes.^49−51^ Al atoms are also relatively straightforward to generate. The product AlF has spectra at accessible wavelengths that are suitable for LIF detection. Conveniently, the incident Al atoms can also be detected by LIF in the same wavelength region. In comparison to the O and F atoms previously used as RAS probes, the recommended van der Waals radius of Al is only some ∼20% larger,^52^ and hence it should remain capable of probing molecular-level surface structure at only marginally lower resolution.

Some previous work has been done on abstraction reactions of Al with gas-phase molecules, including O abstraction from O_2_ and CO_2_.^53−56^ Gas-phase studies of the abstraction of F atoms, however, are more limited, with one notable exception using LIF to study the kinetics of the Al + SF_6_ reaction^57^ at relatively high temperatures. The resulting kinetically determined Arrhenius activation energy was 40 kJ mol^–1^. An activation energy of 25 kJ mol^–1^ for reaction of Al with NF_3_ was also quoted from previous independent, but not publicly accessible, work. Given the known bond energies for SF_5_–F (∼380 kJ mol^–1^) and NF_2_–F (∼240 kJ mol^–1^),^48−51^ it is possible to appeal to the very well-established Evans–Polanyi (sometimes Bell–Evans–Polanyi) principle to estimate activation energies for other reactions.^58^ This principle asserts a linear relationship between activation energy and enthalpy of reaction for a series of “similar” reactions that proceed by a common basic mechanism, such as direct abstraction.^59^ On that basis, the predicted activation energy for F abstraction by Al from a fluoropolymer or fluoroalkyl substituent would be around 60 kJ mol^–1^.

These types of reactions are known to take place in energetic materials based on Al nanoparticles embedded in solid polytetrafluoroethylene (PTFE), where the high barriers are overcome by laser or shock initiation.^60−62^ The implication is that a relatively energetic source of Al atoms will be required for successful RAS-LIF. Fortunately, it is already known that this is achievable via the laser ablation of solid Al targets.^63^ A related approach is used, for example, in the production of AlF for spectroscopic purposes by ablating Al into a carrier gas containing SF_6_, with subsequent cooling by supersonic expansion.^64^

In this work, we apply this new version of the RAS-LIF methodology for the first time to study the composition of fluorine-containing surfaces. We demonstrate the generality by using solid (PTFE) and liquid (perfluoropolyether, PFPE) perfluorinated materials, plus a small set of ILs containing a common fluorinated anion, along with a non-fluorinated blank, for illustrative quantitative measurements. We reiterate, though, that other fluorinated materials could also be studied by this method, with minimal modifications, provided that they have sufficiently low vapor pressures.

## Experimental Section

### Overview

**Figure 1:**
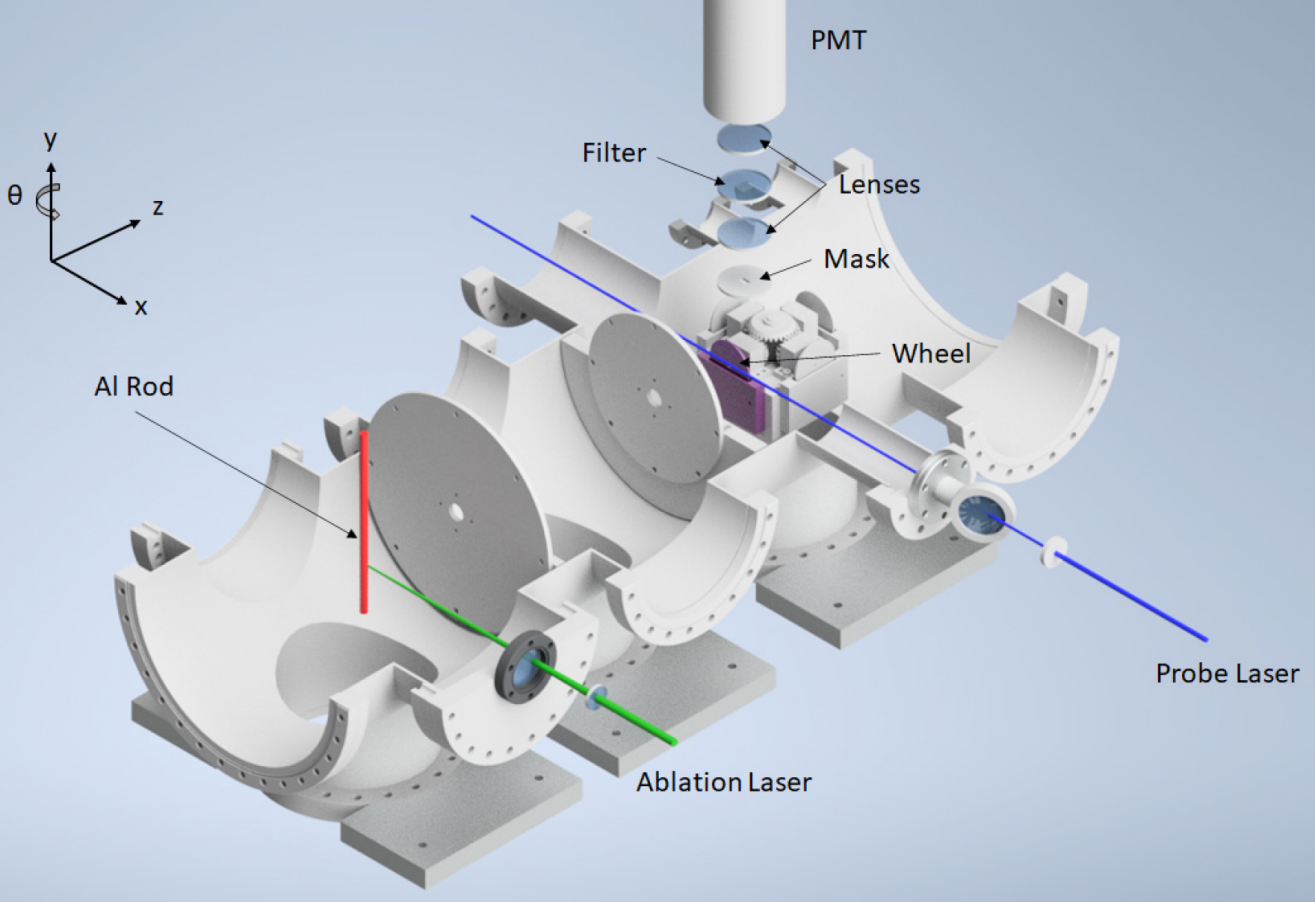
Schematic diagram of the experimental setup.

High-energy Al atoms were created in the source by laser ablation and allowed to fly freely toward the target, which in most experiments was a rotating wheel coated in a fluorinated liquid. The ingoing Al atoms and the reactively scattered AlF products were probed by LIF using a pulsed excitation laser beam that passed a short distance in front of the target surface. The fluorescence was collected and directed onto a photomultiplier tube (PMT). A schematic diagram of the current experimental setup is shown in Figure 1, with a detailed description of each of the key components below. Some preliminary work was performed in a conceptually similar prototype apparatus (see the Supporting Information for details).

### Al Source

The Al atoms were created by laser ablation of an Al rod (>95% purity) using a 532 nm beam produced as the second harmonic of a Nd:YAG laser (Continuum Minilite II), with a pulse length of ∼5 ns and operating at a frequency of 10 Hz. The typically 8.0 mJ/pulse beam was focused onto the rod by using an *f* = 300 mm lens. This created pits ∼350 μm in diameter on the rod, which equated to an average fluence of ∼8.3 J cm^–2^. The laser hit the rod at a point approximately 45° between the entrance window of the laser and the central axis of the chamber. The rod was mounted on an *x*, *y*, *z*, θ manipulator (axes as defined in Figure 1) and was offset from the central chamber axis by approximately its radius (4 mm) to allow the on-axis atoms in the Al plume to travel (along *z*) toward the wheel assembly. The *y* and θ axes of the manipulator were controlled by stepper motors in microstepping modes. The rod was rotated through an angle of θ = 0.45° (corresponding to a distance of ∼30 μm around the circumference of the rod) after every fifth laser shot. Similarly, after the rod had been rotated 355°, it was translated vertically (along *y*) by ∼30 μm. These movements ensured consistency of production of Al atoms; the angles and distances were determined empirically by monitoring the shot-to-shot stability of the Al atom yield as measured by LIF.

The Al plume was emitted over a wide angular range, resulting in the deposition over time of a thin film on the laser-entrance window. The consequent reduction of the effective laser fluence affected the Al yield and its velocity distribution. This was mitigated by the installation of baffles: one (4 mm diameter aperture) immediately adjacent to the laser input window and another (7 mm diameter aperture) 118 mm from it. This ensured that only Al atoms traveling on trajectories between the two apertures could reach the input window, prolonging its usable lifetime. Regular measurements of the laser energy at the rod were performed by translating the rod out of the beam path and detecting the energy exiting the chamber. The entrance laser window was then rotated when the desired laser energy was not achievable. In practice, this was approximately every ∼10^6^ laser shots. The exit window was in the shadow of the rod and, consequently, was not appreciably coated by Al.

The Al atoms traveled 480 mm, passing through two 18 mm diameter apertures before reaching the target. In exploratory measurements, this was a static sheet of solid PTFE, mounted in an optical filter holder. Most quantitative measurements used a liquid-coated wheel. The wheel assembly consisted of four separate wheels (50 mm diameter) and baths to allow for efficient comparative measurements of different samples without breaking vacuum. The continuously refreshed liquid surfaces were created by rotating (30 rpm) the partially immersed wheel in a bath of liquid. The temperature of the liquid was controlled by thermostatic heating. For the measurements reported here, the temperature was set to 47 °C. Further details of the wheel bath assembly can be found elsewhere.^33^

### LIF

The probe beam was produced by a frequency-doubled Sirah Cobra Stretch dye laser (225–228 nm, ∼5 ns FWHM, Coumarin 450, 2400 lines/mm, BBO doubler) pumped by the 355 nm third harmonic of a Nd:YAG laser (Continuum Surelite II) operating at 10 Hz. The probe beam was approximately circular and apertured by an iris to a diameter of 3 mm. Its pulse energy was adjusted to 1 μJ by rotating a λ/2 waveplate relative to a fixed linear polarizer, acting as a variable attenuator. The beam entered and exited the chamber via Brewster angle windows, passing 10 mm in front of the wheel parallel to its surface and vertically displaced by 12.5 mm from its central rotation axis.

To collect the LIF from the Al or AlF, a collection lens (*f* = 40 mm) was placed at its focal length above the centerline of the chamber and centered on the probe laser beam. To discriminate against scattered laser light, a mask was inserted 15 mm below the collection lens. This mask had a rectangular slot that was 10 mm in length along the probe-beam propagation direction and 5 mm in width along the surface normal. A bandpass filter (Edmund Optics #67-870, 228 nm center, 10 nm FWHM, peak transmission 20%) was placed between the collection lens and a second refocusing lens (*f* = 40 mm) to spectrally isolate the fluorescence signal from any stray light. The transmitted light was detected by the PMT (Electron Tubes 9813QB), which was gated by pulsing its high-voltage supply to isolate signals in the vicinity of the probe pulse and to exclude any stray light from the ablation laser. The time-resolved output was recorded by using an oscilloscope (LeCroy HDO4034, 350 MHz) and sent to a data-acquisition computer. The timings of the lasers and triggers were controlled by a delay generator (Quantum Composers 9520).

LIF signals were recorded from ground-state Al(^2^P_1/2_ and ^2^P_3/2_) via transitions to excited ^2^S and ^2^D states^65^ and from electronic ground-state AlF on the AlF(A^1^Π–X^1^Σ^+^) transition.^66^ For the purposes of accumulating excitation spectra or appearance profiles, the desired LIF signal was isolated from residual scattered probe-laser light in software by integrating it over a time gate immediately after the probe pulse. The gate width was set to either 15 or 30 ns for AlF or Al, respectively, reflecting differences in their observed fluorescence lifetimes. A background time gate (60 ns) positioned before the probe pulse was used to correct for any DC fluctuations. When appearance profiles were measured, signals were typically averaged over 50 laser shots per delay between ablation and probe pulses. Five individual appearance profiles were recorded for one sample of liquid, immediately followed by five profiles of the liquid with which it was being compared.

### Materials

**Figure 2:**
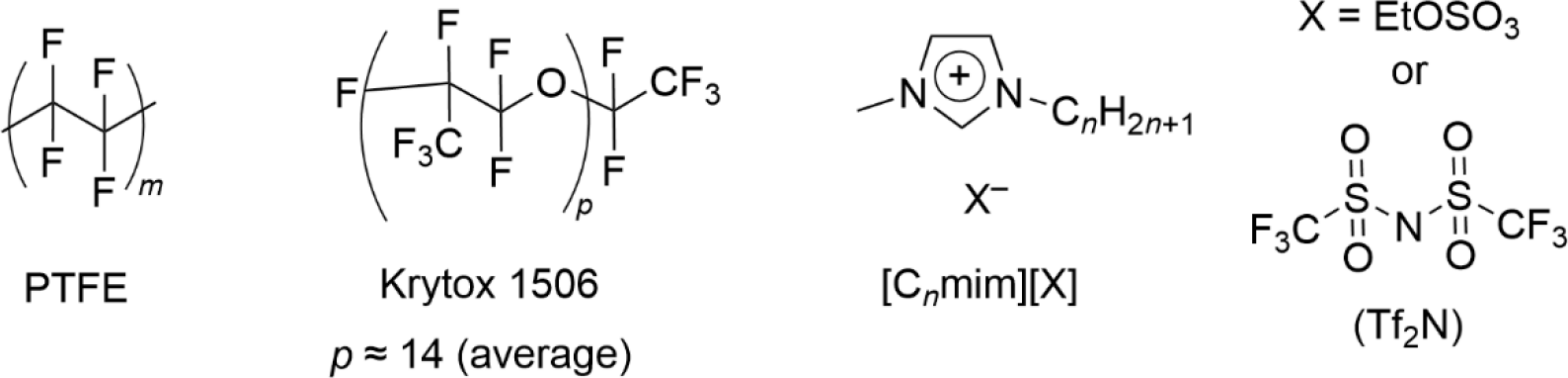
Materials used in this work.

The chemical structures of the materials used are shown in Figure 2. A PTFE sheet (RS Pro) 1.5 mm thick was cut into 50 × 50 mm^2^ squares. The sheet was cleaned in methanol and propan-2-ol to remove any surface contaminants prior to being placed in the vacuum chamber, which was evacuated to a pressure of <10^–7^ mbar for at least 3 h before measurements were performed.

Perfluoropolyether (PFPE; Krytox 1506 (F-[CF(CF_3_)CF_2_O]_14(ave)_-CF_2_CF_3_), DuPont) was housed in a copper bath in our prototype apparatus (as described in the Supporting Information).

The ionic liquids were based on the 1-alkyl-3-methylimidazolium cation with *n* = 2 or 8. The anions were the F-containing bis(trifluoromethylsulfonyl)imide ([Tf_2_N]^−^) and the nonfluorous [EtOSO_3_]^−^ (selected as a suitable blank). Details of the synthesis of the ILs can be found in the Supporting Information.

All ILs were degassed in a separate purpose-built vacuum chamber at a pressure of <10^–6^ mbar for at least 3 h prior to transferring them into the main chamber, where they were subsequently held at <10^–7^ mbar for at least 12 h before measurements were recorded.

## Results

### Al Source Characterization

**Figure 3:**
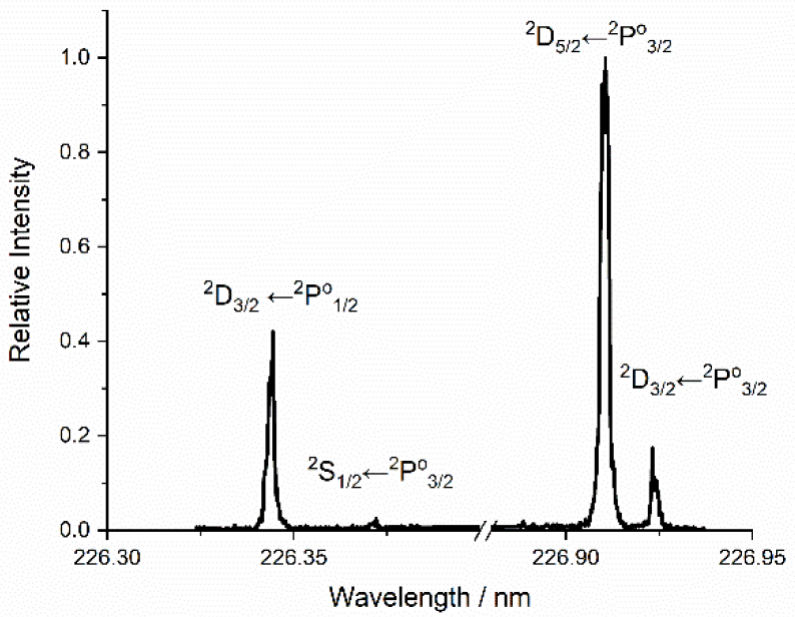
LIF excitation spectrum for ground-state Al atoms at a delay of 50 μs.

The LIF excitation spectrum in Figure 3 shows four transitions from two different spin–orbit states: ^2^D_3/2_ ← ^2^P^o^_1/2_ (226.346 nm), ^2^S_1/2_ ← ^2^P^o^_3/2_ (226.374 nm), ^2^D_5/2_ ← ^2^P^o^_3/2_ (226.910 nm), and ^2^D_3/2_ ← ^2^P^o^_3/2_ (226.922 nm). The overall intensity of all the lines in the spectrum varied strongly and nonlinearly with the ablation laser fluence. There was effectively no observable Al signal for ablation pulse energies below a threshold of ∼3 mJ pulse^–1^, corresponding to a fluence of ∼3 J cm^–2^. We note that this is significantly higher than the 0.69 J cm^–2^ threshold reported by Torrisi et al.;^63^ this may simply reflect, at least in part, different methodologies for estimating the focal spot size. The most intense lines at 226.910 and 226.346 nm can be used to compare the population of the spin–orbit levels of ground-state Al. The relative signals on these two transitions varied only weakly with ablation laser fluence. After accounting for known line strengths,^65^ the ratio of the populations in ^2^P_3/2_ to ^2^P_1/2_ slightly exceeded 2 (which is the relative degeneracy of the two states). These populations are therefore reasonably consistent, for all ablation fluences above threshold, with the Al being produced in a high-temperature plasma where the average thermal energy greatly exceeds the Al spin–orbit splitting, Δ*E*_SO_ = 112 cm^–1^.

**Figure 4:**
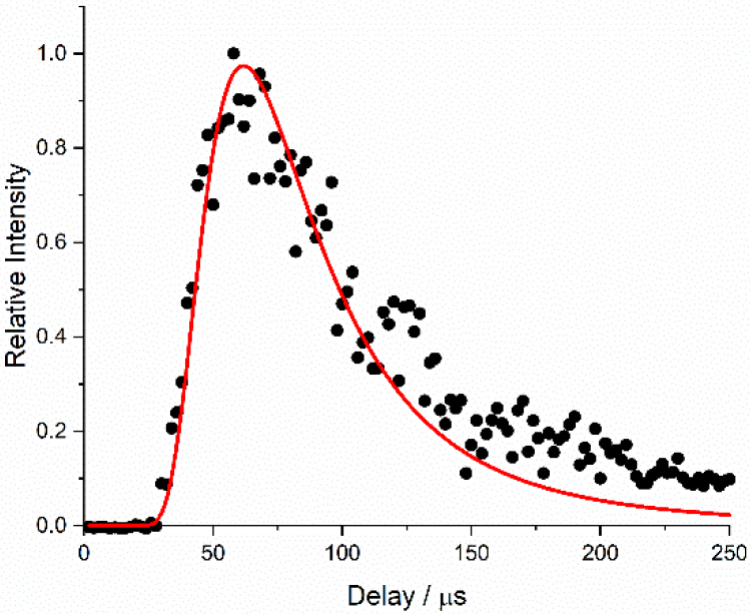
Al atom appearance profiles recorded on the ^2^D_5/2_ ← ^2^P^o^_3/2_ (226.910 nm) transition, produced with an ablation energy of 8 mJ pulse^–1^. Time zero corresponds to the ablation laser pulse. No target surface was present. The best-fit Maxwell–Boltzmann distribution to the first 150 μs is shown in red.

A typical Al atom appearance profile at an ablation pulse energy of 8 mJ, without any target surface present, is shown in Figure 4. The measured time-dependent LIF signal is proportional to Al number density. If the speeds, *v*, in the source are described by a Maxwell–Boltzmann distribution at a defined temperature, *T*, they will have the well-known (normalized) probability density function, *n*_*v*_(*v*):
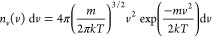
1where *m* is the mass of Al. Assuming that the Al atoms are created instantaneously when the laser pulse hits the Al rod, this may straightforwardly be transformed to predict the distribution that should be observed at a distance, *z*:
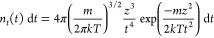
2It was clear from preliminary fitting that the quality of the fit of eq 2 to the data in Figure 4 is good over the rising edge out to delays of ∼120 μs, after which it systematically underpredicts the observations. The deviations are more obvious in measurements with even longer delays (see the Supporting Information). The fit shown in Figure 4 is to the first 150 μs, which yields a temperature of 47000 ± 1000 K. The source of the discrepancy at longer delays is currently unclear; the ablation process may simply not be well-described by a single temperature, or some of the atoms observed at longer delays may not have traveled directly from the source to the observation point without secondary scattering from other surfaces in the chamber.

**Figure 5:**
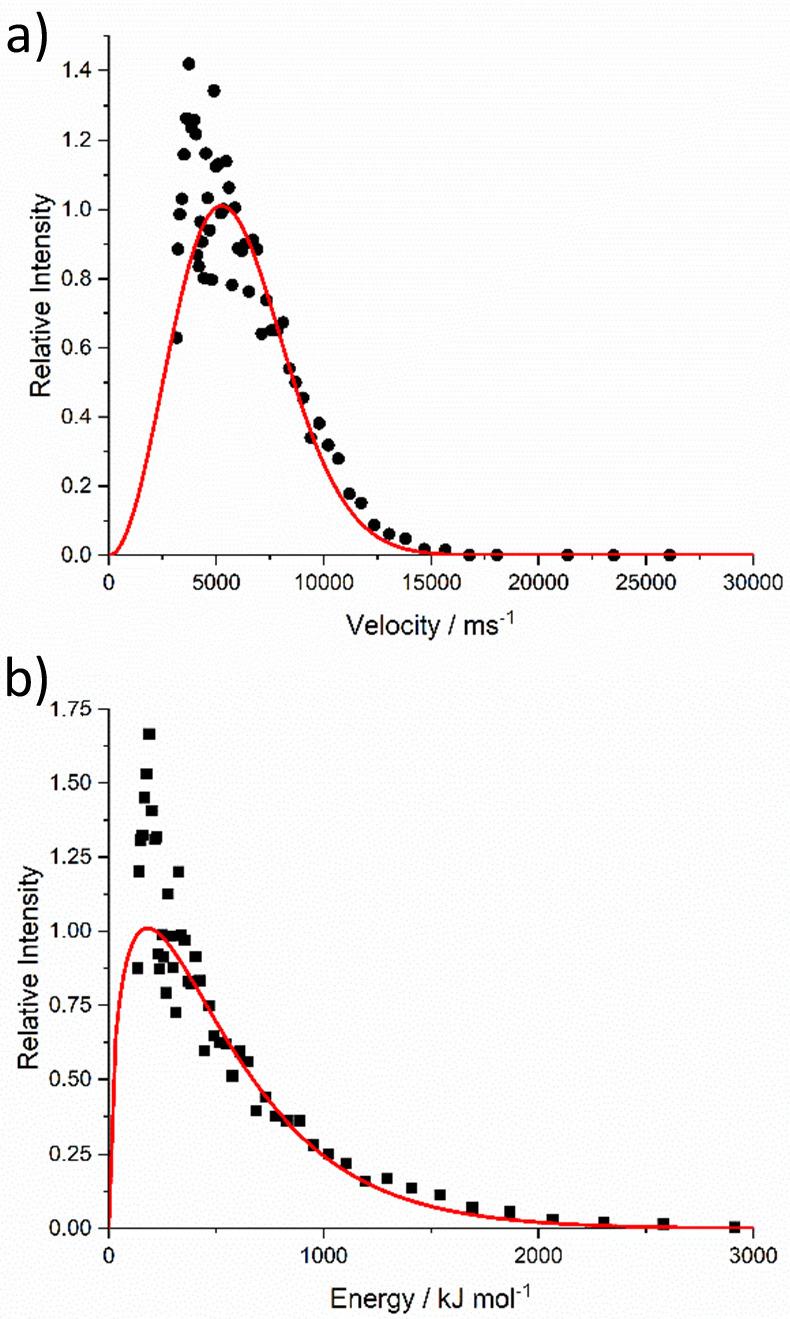
(a) Speed distribution and (b) kinetic energy distribution of the Al atoms derived from the appearance profiles in Figure 4. Red lines are best-fit Maxwell–Boltzmann distributions over the ranges of speed or energy, corresponding to the first 150 μs of the appearance profile. The experimental data are peak-normalized to the fits. Data at slower speeds/lower energies have been binned and averaged to improve visual clarity.

Nevertheless, it is still instructive to transform the observed number-density distribution to the corresponding probability densities for velocity, *n*_*v*_(*v*), or kinetic energy, *n*_*E*_(*E*):
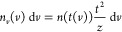
3
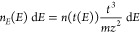
4For the reasons just stated, signals at times longer than 150 μs (and hence slower speeds or lower energies) were neglected in the transformations. The remaining speed or energy components are shown in Figures 5a and 5b, respectively. As would be expected, other than due to the distribution of noise in different regions of the data, they also fit quite well independently to Maxwell–Boltzmann distributions at very similar temperatures (45000 ± 2000 and 44000 ± 2000 K, respectively), as shown. The most-probable speed is ∼5300 ms^–1^; the most-probable energy is ∼187 kJ mol^–1^, and the corresponding mean energy is ∼560 kJ mol^–1^.

### PTFE Measurements

**Figure 6:**
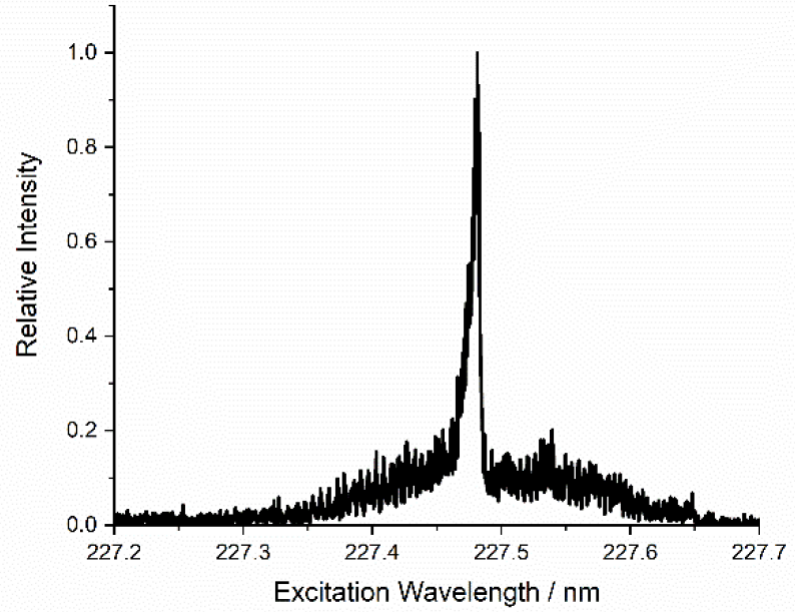
AlF A–X excitation spectrum from the Al reaction with a solid PTFE sample. Delay after ablation = 70 μs; ablation laser pulse energy = 8 mJ/pulse.

PTFE was selected as a suitable solid-surface candidate for the RAS-LIF methodology using Al atoms. As a perfluoropolymer, it will have a high density of fluorine atoms available for abstraction. Strong additional LIF signals were indeed detected on exposure to the Al beam, which were identified as AlF by the characteristic A–X LIF excitation spectrum shown in Figure 6 (see below for more detailed assignments of lines).^66^

The static nature of the PTFE sample meant that the same spot was dosed repeatedly with the Al beam. The deposition of a surface coating, presumably solid Al in some form, was visible to the naked eye as a faint gray metallic sheen that developed during exposure. The AlF LIF signal dropped by 50% within ∼40 min of exposure to the Al source at the 10 Hz repetition rate of the experiment (more details are given in the Supporting Information). Interestingly, this was preceded reproducibly by a smaller initial increase (around 25%) in AlF signal over a period of less than ∼10 min. This may reflect the initial removal of an overlayer that reduces the exposure of F atoms or an increase in reactivity as the morphology of the PTFE surface changes as a result of reaction with Al. We have not yet investigated this in any detail.

### PFPE Measurements

Although the excitation spectrum from the solid PTFE target demonstrated successfully that AlF was being produced from the reaction of Al atoms at its surface, the observed steady decline in the signal made it less convenient for quantitative measurements requiring longer exposure times.

**Figure 7:**
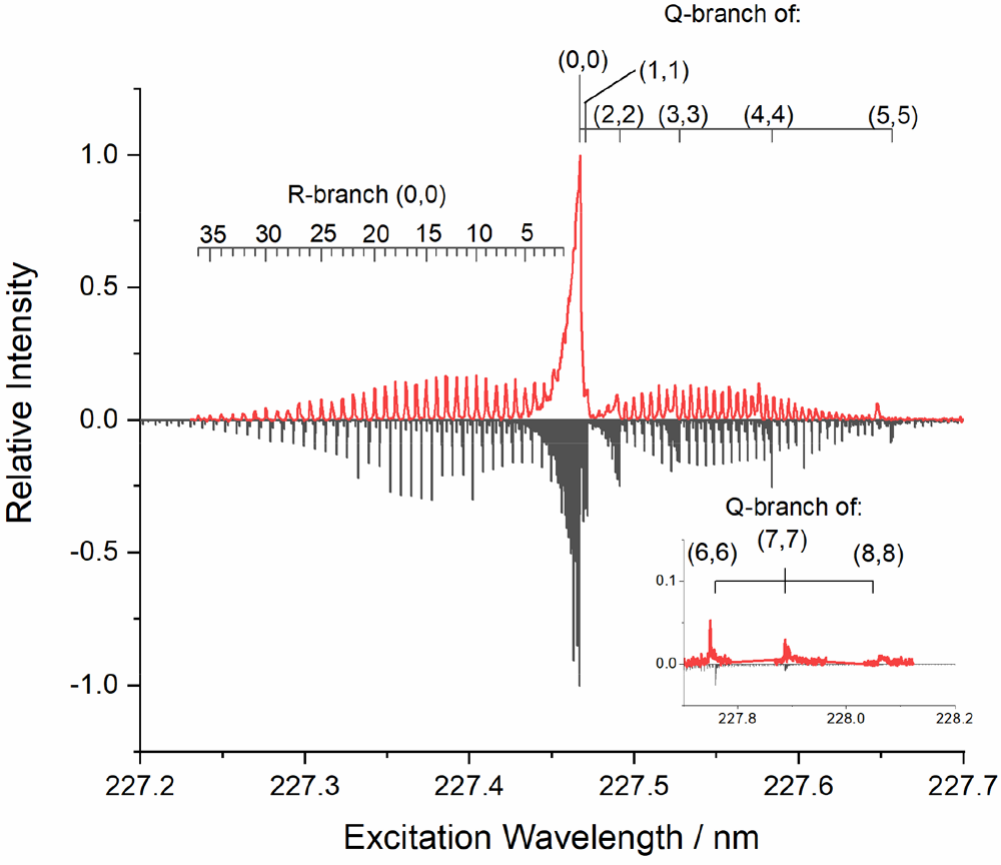
AlF excitation spectrum obtained from the reaction of Al with a liquid PFPE sample in our initial test system (upper trace) and PGOPHER simulation (lower trace) using a rotational temperature of 300 K and a synthesis of several vibrational temperatures up to 1500 K.

Much more consistent AlF signals were detected from the continually refreshed fluorinated liquids. This was first demonstrated in preliminary measurements (see the Supporting Information) using a sample of liquid PFPE. Figure 7 shows a LIF excitation spectrum recorded at the peak of the AlF appearance profile, compared to a simulation generated using PGOPHER.^67−71^ This shows a dominant contribution from AlF(X) *v* = 0 on the (0,0) band, with sufficient resolution in the P and R branches to assess the near-thermal (300 K) rotational distribution. There is also clear evidence of significant vibrational excitation, with Q-branch band heads visible for the diagonal bands (1,1), (2,2), (3,3), etc., up to at least *v* = 8. (The spectrum used to illustrate these features is a weighted synthesis of several vibrational distributions with temperatures up to 1500 K, but it is not intended as a good description of the overall vibrational distribution. The positions of the Q-branch bandheads are progressively less accurately reproduced for higher vibrational levels.)

### Ionic Liquids

**Figure 8:**
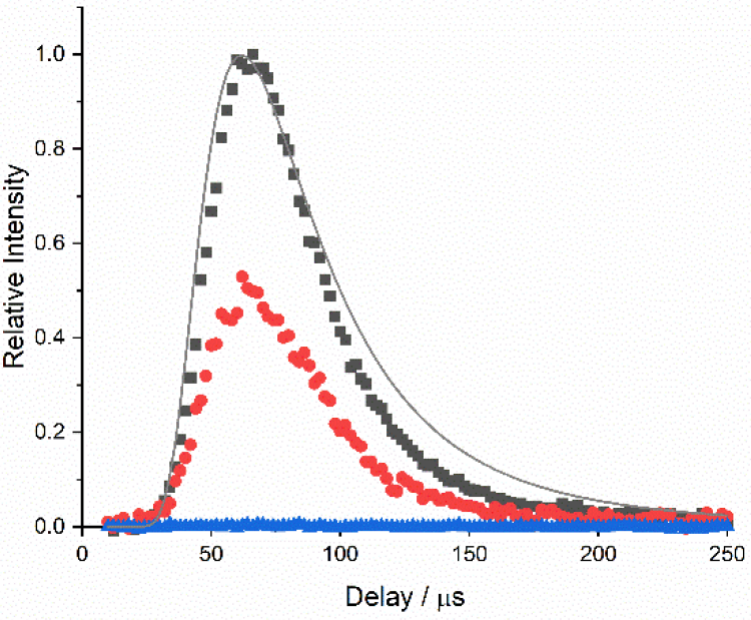
AlF appearance profiles for [C_2_mim][Tf_2_N] (black), [C_8_mim][Tf_2_N] (red), and [C_2_mim][EtSO_4_] (blue), normalized to the peak of the [C_2_mim][Tf_2_N] profile. Signals recorded at the peak of the Q branch of the AlF A–X(0,0) band. Time zero corresponds to the ablation laser pulse. The best fit to the incident Al profile (gray line, see Figure 4), normalized to its own peak, has been included for comparison.

To demonstrate that there is a relationship between the AlF yield and the nature of the liquid sample, appearance profiles were recorded for a small illustrative set of related ionic liquids. The [Tf_2_N]^−^ anion was selected as a suitable source of F atoms through its −CF_3_ groups (see Figure 2). Profiles, recorded at the peak of the AlF A–X(0, 0) Q branch bandhead, from [C_2_mim][Tf_2_N] and [C_8_mim][Tf_2_N] are shown in Figure 8, where they are compared with a profile recorded from the nonfluorous liquid [C_2_mim][EtSO_4_]. AlF yields, taken to be proportional to the integral area of the profile from 20 to 200 μs, were calculated relative to [C_2_mim][Tf_2_N]. They were found to be 0.52 ± 0.05 and 0.001 ± 0.005 for [C_8_mim][Tf_2_N] and [C_2_mim][EtSO_4_], respectively. Three sets of independent measurements were performed for each liquid, and the quoted errors represent the standard error of the mean yield. The reproducibility of the measurements was very good, with very little change in the measured AlF appearance profiles recorded on the same day and only small changes in absolute signal sizes when recorded on different days (see the Supporting Information for details). No spurious signal developed when studying [C_2_mim][EtSO_4_], nor any measurable changes in the relative AlF yields from the other liquids, over a period of a number of weeks, during which the liquids were in the chamber simultaneously under operating conditions.

## Discussion

This work has demonstrated proof-of-principle measurements for the use of high-energy Al atoms as an RAS probe of F atom exposure at surfaces. The results show that it is possible for F atoms to be abstracted from the surfaces of both solid and liquid fluorine-containing materials. The current approach is confined to low-vapor-pressure materials, but in principle, as has been shown recently for related gas–liquid scattering experiments, the scope could be extended to higher-vapor-pressure liquids through the use of liquid microjets.^72−76^ We note in passing that Al may be capable of abstracting other atoms from the IL samples. On the assumption of an S=O bond strength similar to that in SO_2_ (∼550 kJ mol^–1^), reaction with the sulfonyl group to form AlO (bond energy 510 kJ mol^–1^) is estimated to be mildly endothermic. Formation of AlH from reaction with H–C sites (typical bond energies 400–420 kJ mol^–1^) in the cation is significantly more endothermic due to the relatively weak Al–H bond (285 kJ mol^–1^).^49,50^ However, both reactions are energetically feasible, given the high kinetic energies of the Al atoms. We have not yet attempted to detect these species or any of the more complex Al-containing molecules that are conceivable products based on the constituent elements, not all of which will be amenable to LIF.

From a practical perspective, the null AlF signal obtained from the non-fluorinated liquid [C_2_mim][EtSO_4_] is equally important. This demonstrates that there is no AlF being generated in the source, which could conceivably scatter inelastically from the surface and have the appearance of a reactive product. It also shows that no measurable cross-contamination occurs between liquids, despite being present together in the chamber for lengthy periods. This further confirms that unlike solid samples, the liquids are not significantly modified by exposure to the Al source on these time scales with the current duty cycle. This is presumably due to the dilution back into the bulk liquid of the very small fraction of the sample that is exposed at the surface and chemically altered on each laser shot.

A significant factor in the choice of [C_2_mim][Tf_2_N] and [C_8_mim][Tf_2_N] for this work was their relative simplicity, with F atoms present only in the common [Tf_2_N]^−^ anion, combined with the availability of previous related measurements against which to compare the new methodology. The AlF RAS-LIF yields here (see Figure 8) are in a ratio of 0.52 ± 0.05 between long and short alkyl chains, which is in quantitative agreement with the value of 0.52 ± 0.07 for the corresponding F atom peaks in LEIS measurements by Villar-Garcia et al.^77^ They excluded the possibility that the differences are explained by changes in molar volume alone, which could account for only a factor of ∼1.4 increase in the proportion of the anion in [C_2_mim][Tf_2_N] versus [C_8_mim][Tf_2_N]. Thus, the proposed explanation is that the longer octyl chains occupy a larger fraction of the surface area than the ethyl chains, reducing the accessibility of F atoms in the anion. This phenomenon had been demonstrated in our own previous OH RAS-LIF work,^32,34^ which showed that there is a much greater secondary-H exposure at the surface of [C_8_mim][Tf_2_N] than the (immeasurably low) value for [C_2_mim][Tf_2_N]. However, even for octyl chains, it was inferred that the surface is not completely saturated with alkyl groups because the OH RAS-LIF signal continued to increase at longer chain lengths. The LEIS observations provide independent support for the assertion that some of the surface is occupied by anions. This had also been concluded from earlier DRS measurements^24,78^ and is consistent with molecular dynamics simulations by Pensado et al.^79,80^ and subsequently by others, including ourselves.^29,36,39,44^ However, as Lovelock and co-workers have noted,^15^ this contradicts a number of other earlier studies, including e.g. MAES and NICISS measurements from which it had been concluded that anions were not present at the outer surface for longer chain lengths comparable to C_8_.^3,18−22,81^ The new AlF RAS-LIF results here clearly substantially strengthen the argument in favor of there being significant surface exposure of anions in these liquids. Moreover, the fact that the F atoms are abstractable by Al is consistent with the consensus from a range of techniques that the most probable orientation of the [Tf_2_N]^−^ ion is with its CF_3_ groups pointing toward the vacuum.^6,13,15,80^

The success in these initial experiments shows the potential power of this method, and so further development of the technique is warranted to establish its full potential. Even for closely related materials, as in the limited range of IL examples presented here, more detailed investigations of the shapes of AlF appearance profiles are necessary to ensure that density-flux effects do not bias quantitative relative yields. Likewise, possible empirical corrections for variations in the AlF rovibrational state distributions as characterized by LIF excitation spectra, which may vary with the appearance time, would need to be established.

For more chemically distinct materials containing F in different chemical environments, there are very likely to be substantial variations in the excitation function (i.e., AlF yields as a function of Al kinetic energy). This aspect could be investigated by tuning the kinetic energy of the incident Al atoms, either crudely by simply varying the ablation laser fluence or more precisely, in principle, by mechanical chopping of the Al beam. It is not straightforward to predict what the effects of kinetic energy would be on the overall reaction probability or on the resulting AlF kinetic and internal energy distributions. On the positive side, if they could be understood, or again at least calibrated, this may conceivably provide a route to selective F atom detection from different functional groups.

The kinetic energy is also likely to be a key factor controlling the penetration depth of the Al atoms into the liquid and hence is related to the degree of surface sensitivity. In our previous OH RAS-LIF and preceding related work, the photolytically generated O(^3^P) atoms had relatively low kinetic energies (mean of 15.8 kJ mol^–1^).^32^ This made it *a priori* unlikely that they would penetrate deeply into the liquid while sustaining sufficient kinetic energy to surmount the barriers for H abstraction from typical C–H bonds.^82^ (It also made them preferentially sensitive to the weaker secondary (or tertiary, if present) C–H bonds.^32,34,83^) This conclusion was supported indirectly by the observed OH translational and internal-state distributions, which indicated the OH had not been thermalized before escaping the liquid.^35,84−90^ Previous related experiments confirmed the limited penetration depth of such low-energy O(^3^P) atoms into SAMs through selective isotopic labeling of positions on their alkyl chains.^42,43^ The significantly higher, hyperthermal kinetic energies of O(^3^P) (∼500 kJ mol^–1^)^32^ or F(^2^P) (384 kJ mol^–1^)^39^ in related RAS-MS experiments by Minton and co-workers made them capable of abstracting H atoms from a wider range of C–H sites, but a large fraction of the observed OH or HF yield was still associated with direct, impulsive scattering at the surface.^32,35−39,45−47^

It remains to be seen whether this is also true of the Al atoms here, with most-probable energies somewhat lower than those in the relatively narrow distributions of Minton and co-workers but with comparable mean energies and a tail that extends to significantly higher energies (see Figure 5b). There is some preliminary evidence of dynamically determined nascent AlF vibrational excitation but not of rotation in the LIF excitation spectrum in Figure 7. This aspect should be explored further in future work. Furthermore, the delay between rising edges of the incident Al and scattered AlF profiles (see Figure 8), as estimated at their midpoints on an expanded scale, is around 5 μs. These Al atoms have speeds of order 10000 m s^–1^, so the time taken for them to cover the remaining distance of 10 mm to the liquid surface is only ∼1 μs. The remaining ∼4 μs is the return-trip time for the AlF from the surface, but this still corresponds to a substantially superthermal speed of ∼2500 m s^–1^. In comparison, the most probable speed for AlF with a thermal distribution at the liquid surface temperature (320 K) is 340 m s^–1^; the corresponding delay in the appearance profile would be 30 μs. Clearly, the fastest AlF observed must be formed in a direct, impulsive scattering (IS) process. Some momentum exchange is expected for a surface of finite effective mass, but the fastest AlF has not undergone the additional loss of initial kinetic energy, nor of the relevant proportion of the reaction exothermicity, to reach the opposite, thermal desorption (TD) limit.^35,76,91^ This is strong evidence that at least some of the AlF is formed at the outer surface of the liquid and not at depths where the thermalization process would be rapid, as has been widely argued for previous RAS studies as noted above.^32,35−39,45−47,84−90^

Although higher kinetic energies obviously make ballistic penetration of the liquid surface more probable, they do not, in themselves, necessarily mean that the observations will not be surface sensitive. This will still be true if either the identified product can only be formed close to the surface or its escape is suppressed from greater depths. For example, in related work by Qin et al.^92^ on isotopically labeled SAMs using 5–20 eV O^+^ ions as the projectile, the observed OH^–^/OD^–^ signals only result from reaction with the three terminal carbons on the alkyl chain. This is presumably because of the combination of a double charge-exchange process and abstraction of an H (or D) atom, which together act to suppress any yield from greater depths. Related arguments apply in MAES (20 eV, He*)^17,18^ and even more extremely at substantially higher kinetic energies in other methods based on ion scattering such as LEIS (3 keV He^+^)^14−16^ or HRBS (400 keV He^+^).^11−13,93^ In these techniques, the surface sensitivity is derived from a combination of the high probabilities of both charge exchange and inelastic energy loss within the liquid. This is also the basis of surface sensitivity in ARXPS, which relies on the efficient inelastic scattering of escaping photoelectrons.^5,7−10^

There is clearly a general correlation here between the Al production in the ablation process and AlF yield from the target. As yet, however, we have not verified definitively that the AlF is produced in reactions of ground-state Al atoms because ablation can also be expected to produce some proportion of ions and metastable neutral species.^63^ Using a similar type of ablation source, Torrisi et al.^63^ observed Al^+^, Al^2+^, and Al^3+^ ions as well as the neutral Al species. The ions were found to have peak kinetic energies higher than those of the neutrals and higher creation thresholds. They also had progressively narrower angular distributions, more strongly directed along the surface normal. This should mean they are discriminated against to some extent in our experimental geometry, but whether they make any contribution to the AlF yield could be confirmed directly in future work by applying suitable deflection voltages to prevent them from reaching the target. Similarly, at least in principle, the presence of metastable Al species and their correlations with AlF yields could be investigated spectroscopically.

From a purely operational standpoint, the nature of the reactive projectile does not actually matter if the desired objective was simply to develop an empirical surface analytical probe for F atoms. It is obviously fundamentally interesting, however, and a proper mechanistic understanding will clearly enable the method to be developed on a rational basis. We look forward to future work in which we can more fully characterize and begin to apply this promising new technique.

## Conclusions

Translationally hot Al atoms produced in a laser ablation source have been shown to be capable of abstracting F atoms from fluorinated solid and liquid surfaces. Gas-phase AlF yields detected by laser-induced fluorescence are correlated to the expected degree of surface exposure of fluorinated groups in a small, illustrative set of ionic liquids. This may provide the basis of a new variant of the RAS-LIF method for the quantitative measurement of surface composition.

## Supporting Information Available

IL synthesis and characterization; Al appearance profile; characterization of AlF signals from PTFE; description of initial setup used for PFPE measurements; AlF signal reproducibility from ILs (PDF)
