# Supplementary material for: Superthermal Al Atoms as a Reactive-Atom Probe of Fluorinated Surfaces
Source: J Phys Chem A. 2023 Jun 23;127(26):5580–90. doi: 10.1021/acs.jpca.3c02167 (PMC10331727; doi:10.1021/acs.jpca.3c02167)
Supplement: Supplementary file 1 — jp3c02167_si_001.pdf [file jp3c02167_si_001.pdf]

Superthermal Al atoms as a reactive-atom probe of fluorinated surfaces

Paul D. Lane†, Thomas Gstir‡, Simon M. Purcell†, Michal Swierczewski†, Naomi S. Elstone§, Duncan W. Bruce§, John M. Slattery§, Matthew L. Costen† and Kenneth G. McKendrick†.*

† Institute of Chemical Sciences, School of Engineering and Physical Sciences, Heriot-Watt University, Edinburgh, EH14 4AS, UK

‡ Institut für Ionenphysik und Angewandte Physik, Universität Innsbruck, Innsbruck, Austria

§ Department of Chemistry, University of York, Heslington, York, YO10 5DD, UK

Supplementary Material

Contents

1. IL synthesis and characterisation 2
   1.1. Synthesis of [C_2MIM][Tf_2N] 2
   1.2. Synthesis of [C_2MIM][$EtSO_4$] 2
   1.3. Synthesis of [C_8MIM][Tf_2N] 2
2. Al Appearance Profile 3
3. Characterisation of AlF signals from PTFE 4
4. Description of Initial Apparatus used for PFPE Measurements. 6
5. AlF signal reproducibility from ILs 7
6. References 7

1. IL synthesis and characterisation

1.1. Synthesis of $[C_2MIM][Tf_2N]$

$[C_2MIM]Br$ was prepared according to the literature method.[1] Distilled 1-methylimidazole (91.7 g, 89.0 mL, 1.12 mol) was added dropwise to a flask containing an excess of freshly distilled 1-bromoethane (133.8 g, 92.0 mL, 1.23 mol). The mixture was stirred for 15 min at 50 °C, until the initial turbidity disappeared, and then for 2 h at 70 °C. Upon cooling to 0 °C, a white solid formed, which was ground in a glovebox. The solid was treated with ethyl acetate (20 mL) and stirred at −10 °C for 1 h under nitrogen. After removing the solvent via cannula filtration, the white solid was dried in vacuo at 60 °C for 6 h (185.7 g, 87% yield). 1H NMR (400 MHz, dmso-d6, 293 K), δ (ppm): 9.29 (s, 1 H), 7.87 (m, 1 H), 7.78 (m, 1 H), 4.24 (q, 3J = 7.3 Hz, 2 H), 3.90 (s, 3 H), 1.44 (t, 3J = 7.3 Hz, 3 H).

A solution of $[C_2mim]Br$ (20.0 g, 0.11 mol) in deionised water (150 mL) was treated with a solution of Li[Tf2N] (53.2 g, 0.18 mol) in deionised water (150 mL). The biphasic system was stirred overnight at room temperature. An aqueous extraction (4 × 200 mL) was performed to remove the lithium halide until no precipitation of AgBr occurred in the aqueous phase upon addition of $AgNO_3$ solution. The colourless oil was then dried in vacuo at 50 °C (19.21g, 79% yield). ^{1}H NMR (400 MHz, acetone-d6, 293 K), δ (ppm): 9.01 (s, 1H), 7.75 (m, 1H), 7.69 (m, 1H), 4.39 (q, 2H), 4.04 (s, 3H), 1.56 (t, 3H). Elemental analysis: %C 24.36 (theoretical: 24.56), %H 2.85 (theoretical: 2.83%), %N 10.64 (theoretical: 10.74)

1.2. Synthesis of $[C_2MIM][EtSO_4]$

$[C_2MIM][EtSO_4]$ was prepared according to the literature method.[2] Diethyl sulfate (8mL, 0.062 mol) was added dropwise to a solution of 1-methylimidazole (5 mL, 0.063 mol) in dry toluene under inert conditions. Slow addition was required to ensure heat generation was controlled. The solution was stirred under inert conditions for 2 days and a biphasic mixture formed. The top layer was removed via cannula transfer and the product dried under vacuum for 11 days at elevated temperatures between 55 and 70 ^{o}C to ensure removal of solvent and excess 1-methylimidazole (12.93g, 88% yield). ^{1}H NMR (400 MHz, acetone-d6, 293 K), δ (ppm): 9.36 (s, 1H), 7.81 (m, 1H), 7.73 (m, 1H), 4.36 (q, 2H), 4.00 (s, 3H), 3.87 (q, 2H), 1.50 (t, 3H), 1.12 (t, 3H)

1.3. Synthesis of $[C_8MIM][Tf_2N]$

$[C_8MIM]Br$ was prepared following the literature method.[1] Distilled 1-methylimidazole (64 mL, 0.75 mol) was added to dried and distilled 1-bromoctane (174.93g, 0.91 mol) in dry toluene under inert atmosphere. The reaction was heated to 60°C overnight, the toluene removed and the product dried under vacuum at elevated temperature (up to 75°C) for 10 days (198.54g, 96% Yield). ^{1}H NMR (400 MHz, acetone-d6, 293 K), δ (ppm): 10.23 (s, 1H), 7.94 (m, 1H), 7.87 (m, 1H), 4.45 (t, 2H), 4.10 (s, 3H), 1.94 (quint ,2H), 1.29 (m, 10H), 0.86 (t, 3H)

$[C_8MIM]Br$ in dichloromethane (50g, 0.18 mol) was added to a solution of $Li[Tf_2N]$ (68g, 0.24 mol) in deionised water and stirred for 2 days at room temperature. An aqueous extraction (3 x 200mL) was performed to remove lithium halide from the organic phase, once the aqueous layer tested negative for halides (no precipitation of AgI on addition of $AgNO_3$) twice consecutively the DCM was removed on a rotary evaporator and the IL dried under reduced pressure at 60°C (78.22 g, 91% yield). ^{1}H NMR (400 MHz, acetone-d6, 293 K), δ (ppm): 8.94 (s, 1H), 7.71 (m, 1H), 7.65 (m, 1H), 4.33 (t, 2H), 4.01 (s, 3H), 1.91 (quint, 2H), 1.29 (m, 10H), 0.85 (t, 3H)
Elemental analysis: %C: 35.2 (calc 35.37), %H 4.75 (calc 4.88), %N: 8.71 (calc 8.63)

2. Al Appearance Profile

Figure S1 shows the appearance profile for Al $^2P^o_{3/2}$ to a final delay of 1 ms together with the fitted Maxwell-Boltzmann distribution described in the main text. Al atoms are clearly detected at low levels at long delays that are inconsistent with the fitted M-B distribution. A flight time of 1 ms over the ablation to probe-laser-beam distance of 470 mm corresponds to a speed of 470 ms^{-1}, approximately that expected for a room temperature sample of Al. We therefore propose that these late-time Al atoms are the result of secondary collisional thermalisation of the Al with chamber surfaces, combined with the additional path length to reach the probe region implied by wall collisions. As these delay times are much later than the observed appearance times of the AlF produced at the surface, we have not presented them in the main text.

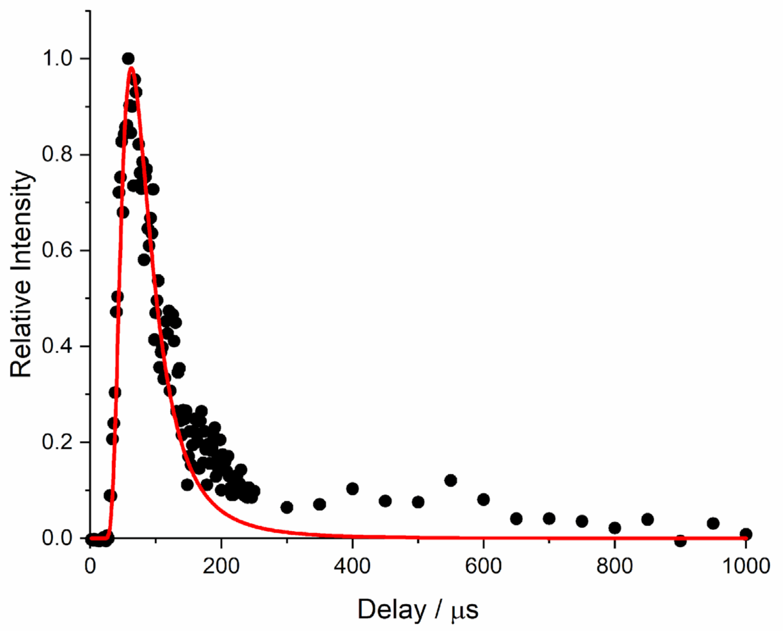

Fig S1: Aluminium appearance profiles for the $^2D_{5/2} \leftarrow {}^2P^o_{3/2}$ (~226.91 nm) line produced with an ablation energy of 8 mJ/pulse. No target surface was present.

3. Characterisation of AlF signals from PTFE

The PTFE sheet was exposed to the incident Al plume at a distance of 480 mm from the source, and sequential AlF appearance profiles were recorded, with 10 laser shots per delay time. These appearance profiles were repeated every 10 minutes, with selected profiles shown in Fig S2. The amplitude of the appearance profiles clearly initially increases, and then decreases to essentially zero through this sequence. Figure S3 shows the overall production of AlF as a function of surface exposure time, calculated from integration of the appearance profiles over a delay of 40 to 200 µs. The initial increase in AlF in the first 10 minutes is attributed to the presence of surface contamination inhibiting the abstraction of F atoms, followed by a rapid reduction of the AlF signal as an Al overcoat is deposited on the surface, with a half-life of approximately 40 minutes.

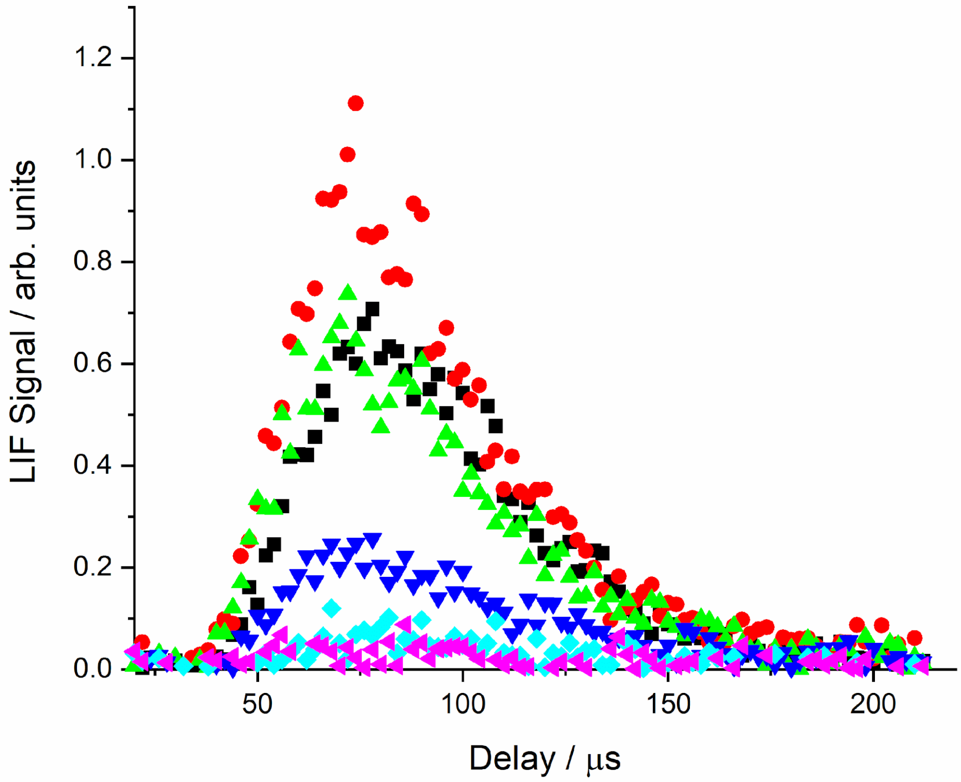

Figure S2: AlF appearance profiles upon exposure to Al for: 0 mins (black), 10 mins (red), 30 mins (green), 60 mins (blue), 120 mins (cyan) and 180 mins (purple).

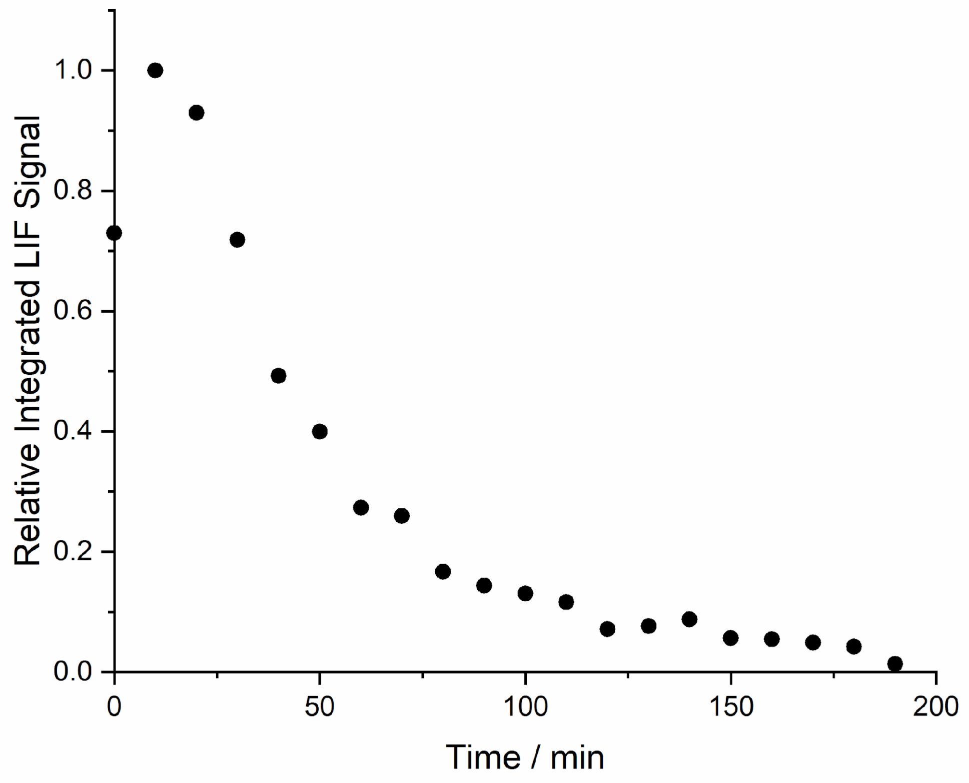

Figure S3: Relative AlF yield as a function of exposure time to the aluminium source.

4. Description of Initial Apparatus used for PFPE Measurements.

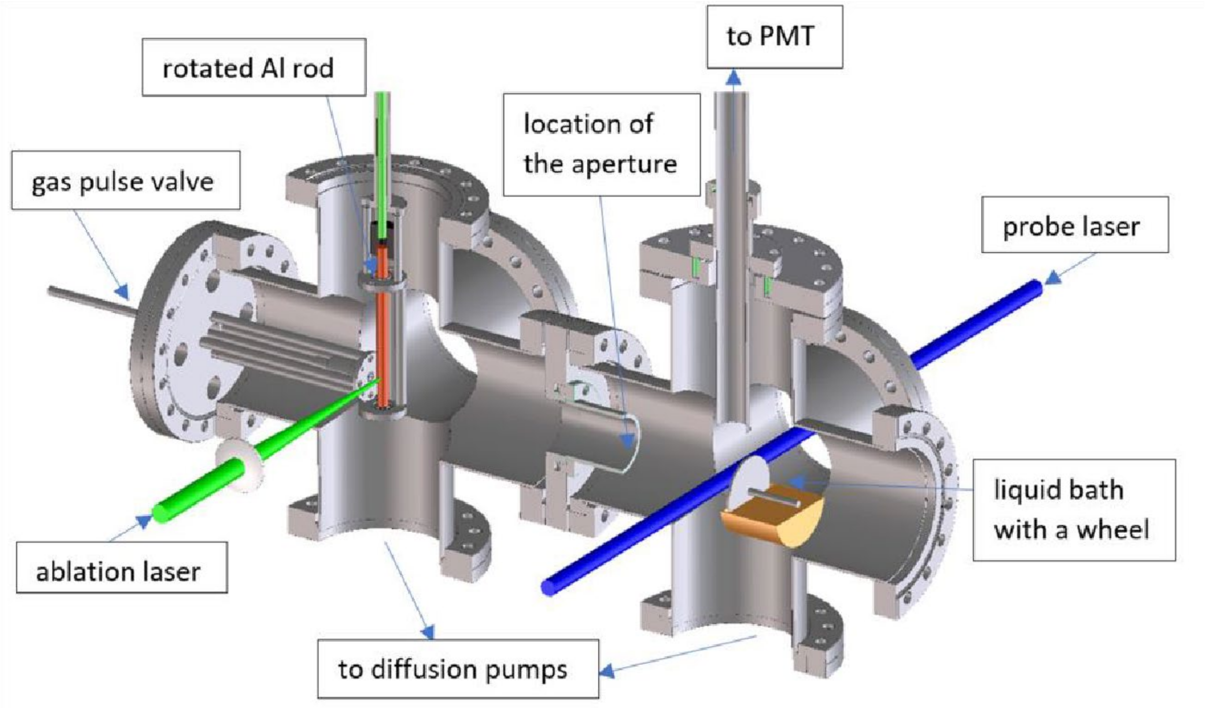

Figure S4: A schematic diagram of the initial experimental setup

The initial apparatus was a simplified version of that described in the main text. A schematic diagram is shown in Fig S4. The Al rod and associated rotation/translation mechanisms were identical, with the only differences lying in the ablation laser, and focussing lens. A Continuum Surelite II-10 Nd:YAG laser was used at the source of 532 nm laser pulses. This has a higher pulse energy than the Minilite used in the main paper. The smaller window-rod distance was smaller than in the new apparatus, allowing an $f = 250$ mm lens to be used. These factors combined allowed a higher laser fluence to be obtained, and as a consequence the data shown in Fig. 7 of the main paper was obtained using an ablation laser fluence of ~ 30 J cm^{-2}. The more compact chamber had a shorter Al flight path (216 mm) from the source to the wheel, which only passed through a single aperture. The distance from the probe beam to the wheel was 6 mm. The liquid surface was created using a single wheel/bath assembly at ambient temperature,[3] with no active temperature control and restricted ability to make comparative measurements of AlF yields from different liquids. The probe laser and LIF detection, timing and data acquisition system were as described in the main paper.

5. AlF signal reproducibility from ILs

Figure S5 shows two panels, each with five individual appearance profiles of AlF from [C_2mim][Tf_2N] recorded at 50 shots per point. No scaling or other renormalization has been applied to any of these profiles. Those in each panel were recorded sequentially in a single measurement session, with approximately 1 hour total measurement time, demonstrating the excellent short-time reproducibility of the experimental signals. The profiles in the two panels were recorded on different days, and hence demonstrate the also excellent day-to-day reproducibility. This includes the effects of daily optimisation of the Al atom source, e.g. position of the rod relative to the ablation laser focus, as well as the setting of the probe laser energy and wavelength. The remaining small differences in the day-to-day measurements are easily corrected by normalising to the signal from a reference liquid sample, as previously demonstrated in our RAS-LIF experiments using O(^{3}P) as the reactive atom.

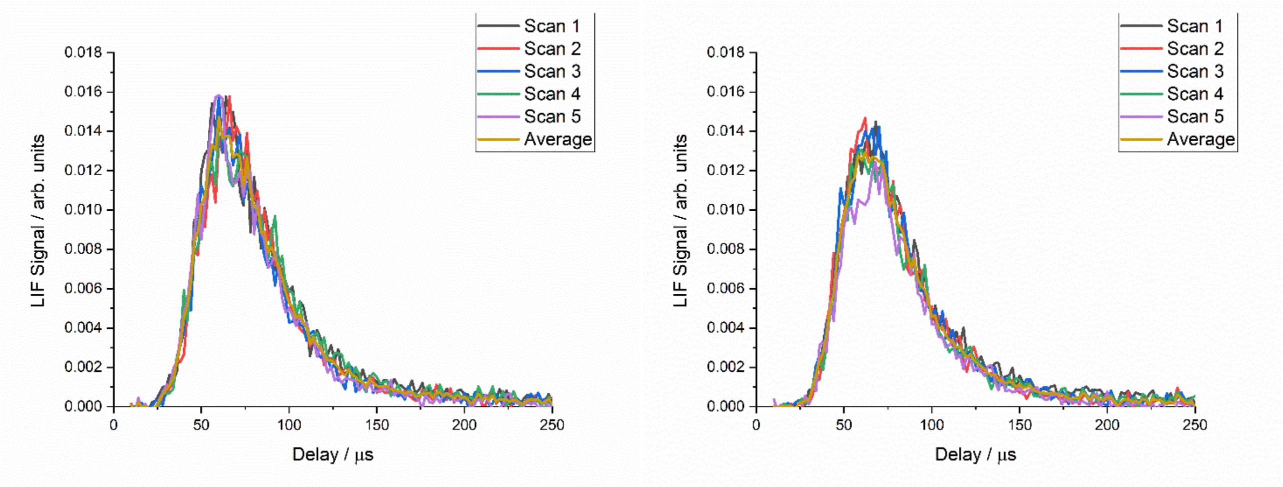

Figure S5: Individual appearance profiles of AlF from [C_2mim][Tf_2N], in two separate sequences of measurements, taken on different days.

6. References

(1) Cabry, C. P.; D'Andrea, L.; Elstone, N. S.; Kirchhecker, S.; Riccobono, A.; Khazal, I.; Li, P. X.; Rogers, S. E.; Bruce, D. W.; Slattery, J. M. Small-angle neutron scattering from mixtures of long- and short-chain 3-alkyl-1-methyl imidazolium bistriflimides. *Phys Chem Chem Phys* **2022**, *24* (26), 15811-15823. DOI: 10.1039/d2cp01528e.

(2) Holbrey, J. D.; Reichert, W. M.; Swatloski, R. P.; Broker, G. A.; Pitner, W. R.; Seddon, K. R.; Rogers, R. D. Efficient, halide free synthesis of new, low cost ionic liquids: 1,3-dialkylimidazolium salts containing methyl- and ethyl-sulfate anions. *Green Chem* **2002**, *4* (5), 407-413. DOI: 10.1039/b204469b.

(3) Waring, C.; Bagot, P. A. J.; Raisanen, M. T.; Costen, M. L.; McKendrick, K. G. Dynamics of the Reaction of O(P-3) Atoms with Alkylthiol Self-assembled Monolayers. *J Phys Chem A* **2009**, *113* (16), 4320-4329. DOI: 10.1021/jp8109868.
